# The Role of Congestion Biomarkers in Heart Failure with Reduced Ejection Fraction

**DOI:** 10.3390/jcm12113834

**Published:** 2023-06-03

**Authors:** Michele Correale, Francesco Fioretti, Lucia Tricarico, Francesca Croella, Natale Daniele Brunetti, Riccardo M. Inciardi, Anna Vittoria Mattioli, Savina Nodari

**Affiliations:** 1 Cardiology Unit, Policlinico Riuniti University Hospital, 71100 Foggia, Italy; michele.correale@libero.it (M.C.); lucia.tricarico.lt@gmail.com (L.T.); natale.brunetti@unifg.it (N.D.B.); 2 Cardiology Section, Department of Medical and Surgical Specialties, Radiological Sciences and Public Health, University of Brescia, 25123 Brescia, Italy; franc.fioretti@gmail.com (F.F.); riccardo.inciardi@unibs.it (R.M.I.); 3 Department of Medical & Surgical Sciences, University of Foggia, 71122 Foggia, Italy; fracroella@gmail.com; 4 Department of Surgical, Medical and Dental Morphological Sciences Related to Transplant, Oncology and Regenerative Medicine, University of Modena and Reggio Emilia, 41121 Modena, Italy; annavittoria.mattioli@unimore.it

**Keywords:** biomarkers, congestion, edema, heart failure, HFrEF

## Abstract

In heart failure with reduced ejection fraction, edema and congestion are related to reduced cardiac function. Edema and congestion are further aggravated by chronic kidney failure and pulmonary abnormalities. Furthermore, together with edema/congestion, sodium/water retention is an important sign of the progression of heart failure. Edema/congestion often anticipates clinical symptoms, such as dyspnea and hospitalization; it is associated with a reduced quality of life and a major risk of mortality. It is very important for clinicians to predict the signs of congestion with biomarkers and, mainly, to understand the pathophysiological findings that underlie edema. Not all congestions are secondary to heart failure, as in nephrotic syndrome. This review summarizes the principal evidence on the possible roles of the old and new congestion biomarkers in HFrEF patients (diagnostic, prognostic, and therapeutic roles). Furthermore, we provide a description of conditions other than congestion with increased congestion biomarkers, in order to aid in reaching a differential diagnosis. To conclude, the review focuses on how congestion biomarkers may be affected by new HF drugs (gliflozins, vericiguat, etc.) approved for HFrEF.

## 1. Introduction (Congestion in HFrEF)

Heart failure (HF) is a complex syndrome consisting of symptoms (e.g., breathlessness and fatigue) usually accompanied by signs (e.g., elevated jugular venous pressure, pulmonary crackles, and peripheral oedema). It is due to a structural and/or functional abnormality of the heart that results in elevated intracardiac pressures and/or inadequate cardiac output at rest and/or during exercise [1].

We can distinguish specific phenotypes based on the assessment of left ventricular ejection fraction (LVEF). Reduced LVEF (i.e., significant reduction in LV systolic function) is defined as ≤40%; those with HF symptoms and signs in the presence of reduced LVEF are designated as HF with reduced ejection fraction patients (HFrEF).

Despite recent advances in HFrEF management (either pharmacological or non-pharmacological), HFrEF remains a highly prevalent disorder with a high mortality rate and is burdened by several hospitalizations for acute decompensated heart failure (ADHF) [1].

ADHF hospitalizations are generally associated with signs and symptoms of congestion [2]. ADHF recurrence is often the consequence of fluid retention, which leads to both systemic and pulmonary congestion [3,4,5]. In HFrEF patients, congestion may be an important target for therapy. Moreover, the lack of congestion resolution during HF hospitalization or the occurrence of residual edema after discharge may be associated with poor outcomes [6]. Increased severity of congestion evaluated by a simple orthodema assessment is related to augmented morbidity and mortality [7].

Systemic congestion is not exclusively related to cardiac congestion, and the simple absence of clinical signs of fluid overload cannot exclude increased left ventricular filling pressures (LVEDP) [8].

We can distinguish two different forms of congestion: the intravascular one if congestion is predominantly present in the vascular system, and the tissue one if it is interstitial. Although most HF patients have a combination of both forms, one phenotype can prevail. Each of these kinds of congestion has a specific pathophysiology and requires a different diagnostic approach.

Peripheral congestion occurs in the form of peripheral edema, with a palpable swelling in the body tissues caused by an expanded interstitial fluid volume. This occurs if the capillary filtration exceeds the amount of fluid conducted out by lymphatic drainage. It is observed in different clinical conditions like HF, renal failure, liver failure, or pathologies affecting the lymphatic system. Edema usually becomes clinically apparent when the interstitial volume exceeds 2.5–3 L.

Assessment of congestion by a simple clinical evaluation remains unsatisfactory. The most commonly used and traditional strategy is clinical evaluation, associated with chest radiography and natriuretic peptide (NP) measurement [9,10].

It is very important for clinicians to predict the presence of congestion with biomarkers and to understand the pathophysiological mechanisms that underlie edema.

There is increased attention not only on the role of congestion biomarkers in recognizing patients at risk of developing HF who are potential candidates for targeted treatments for its prevention, but also on their role in formulating an initial diagnosis and a prognostic stratification.

This review summarizes the principal evidence on the possible roles (diagnostic, prognostic, and therapeutic) of old and new congestion biomarkers in HFrEF patients. Furthermore, we describe conditions other than congestion with increased congestion biomarkers in order to facilitate a differential diagnosis. To conclude, the review focuses on how congestion biomarkers may be affected by new HF drugs (gliflozins, vericiguat, etc.) approved for HFrEF.

## 2. Definition of Biomarkers

A biomarker is a biological molecule found in blood, other body fluids, or tissue and may relate to data obtained from vital parameters (e.g., blood pressure), or from imaging/instrumental tests [11].

Biomarkers can help in identifying disease peculiarities (risk factor or risk marker), disease state (preclinical or clinical), or disease outcome (rate of progression) [12]. Biomarkers can be classified as antecedent biomarkers (assessing the risk of developing a disease), screening biomarkers, diagnostic biomarkers, staging biomarkers (categorizing the disease severity), or prognostic biomarkers (if helpful in predicting the evolution) [13]. They play different roles in diagnosis, prognostic prediction, and assessment of therapy response, like natriuretic peptides in HF or sputum nanoparticles in inflammatory lung disease do [14].

Ideal biomarkers should be precise, standardized, patient-acceptable, easily interpretable by clinicians, sensitive, and highly outcome-specific.

## 3. Congestive Biomarkers

Many researchers have sought out cardiac biomarkers to improve the prediction, diagnosis, and prognosis of HF. Several biomarkers related to different findings in HF pathophysiology have been studied (Table 1) [15,16].

Natriuretic peptides (NPs) are the most known and widely used biomarkers. Brain natriuretic peptide (BNP) and atrial natriuretic peptide (ANP) are neurohormones produced and stored in the heart, both in the atria and ventricles, and released from the heart due to increased end-diastolic wall-stress in response to pressure changes and volume overload.

NPs are synthesized as prohormones and subsequently cleaved into the active hormones BNP and ANP, and into the inactive NT-proBNP and mid-regional proANP (MR-proANP). Circulating BNP and ANP half-lives are relatively short (about 20 min). Plasma endopeptidases and NP receptors remove them from the circulation. NT-proBNP and MR-proANP have longer circulating half-lives (around 90 min), and their clearance is mainly renal [17,18].

Cardiac troponins (cTn) are usually evaluated in patients with acute HF (AHF) to rule out myocardial infarction. However, congestion at the inlet was significantly associated with cTn levels at the time of discharge, implying that the high intracardiac filling pressure and the increased wall stress associated with HF decompensation can induce subclinical myocardial injury [19].

The primary function of troponin levels in HF is to stratify risks. Higher troponin I or T levels at the hospital admission of patients with AHF were associated with lower EF and a higher rate of in-hospital mortality [20,21].

Recently accumulating data suggest cancer antigen-125 (CA-125) as a marker of congestion in HF. The increase in circulating CA-125 concentrations is due to at least two pathophysiological mechanisms that partially overlap [22]. On the one hand, there is the mechanical stress produced by excessive fluid accumulation. This increased stress activates c-Jun N-terminal kinase (JNK) pathways and raises the synthesis of CA-125 [23]. Moreover, there is the activation of the O-glycosylated extracellular domain of CA-125. The result is the release of CA-125 from the actin cytoskeleton of mesothelial cells and its increased concentration in the periphery [24]. On the other hand, there is inflammation. A linkage between CA125 and proinflammatory cytokines, such as tumor necrosis factor (TNF)-α, interleukin (IL)-6, and IL-10, has been described [25]. Venous congestion has been shown to alter the expression of certain models in the endothelium and congested perivascular tissue to the activated state, resulting in an upward regulation of pro-oxidant, proinflammatory, and vasoconstricting factors [26]. Furthermore, inflammatory stimuli worsen fluid overload by affecting the neurohumoral and endocrine systems [27]. Overall, volume overload and inflammation in HF mutually interact, increasing each other’s activity in a bidirectional manner, thus creating a positive feedback loop that leads to elevated CA125 concentrations [28].

CA125 has been demonstrated to be a tool for risk stratification in patients admitted with ADHF and CA125 <23 U/mL identifies a subgroup of patients at low risk of short-term adverse events [29].

CA125 and no NT-proBNP seem to identify patients with AHF, and congestive intrarenal venous flow (IRVF) patterns. The IRVF measured by Doppler ultrasound may be a potential surrogate marker of renal congestion and adverse outcomes in heart failure [30].

Soluble suppressor of tumorigenesis-2 (sST2, also known as interleukin-1 receptor-like 1), the circulating form of the interleukin-33 membrane receptor, is secreted by myocardial cells in response to cardiac overload. Vascular congestion, mechanical stretch, and inflammation stimulate the expression of sST2 [31]. Lungs have been documented as a relevant source of sST2 in HF. sST2 is a powerful predictor of mortality and hospitalization in AHF or chronic HF (CHF) independently from NT-proBNP, hs-troponin T, and LVEF, almost unaffected by age, sex, body mass index, renal function, or ischemic aetiology [32].

The protein galectin-3 is currently gaining interest as an eligible biomarker in cardiac disease. Galectin-3 is a biomarker of fibrosis, inflammation, and oxidative stress. Normally, Gal-3 expression in the heart is low. In the failing heart, Gal-3 is released by activated cardiac macrophages and cardiac fibroblasts [33], taking part in ventricular remodeling [34]. Elevated galectin-3 levels in patients with HFrEF are associated with concomitant RV dysfunction and exercise intolerance [35].

Cholestatic liver injury can be measured by bilirubin, alkaline phosphatase, and gamma-glutamyl transpeptidase levels [36], which have been suggested as possible congestion biomarkers [37]. Moreover, the decreased cardiac output and subsequent low liver perfusion may induce acute hepatocellular necrosis [38]. Increased levels of aspartate aminotransferase, alanine aminotransferase, and bilirubin are found in patients with low cardiac output [39].

Laboratory anomalies of liver function may predict the prognosis of patients with advanced HF, and the assessment of both cardiac and liver function is very important in the management of these patients [40].

Hemoconcentration could be another sign of congestion. Plasma volume may be indirectly estimated by several formulas using hemoglobin and/or hematocrit levels, which seem useful for monitoring congestion and decongestion both in acute and chronic settings [41]. Several other biological parameters that are routinely evaluated in patients with HF, such as serum protein, albumin, hemoglobin, and hematocrit, have been correlated with prognosis and proposed as alternative markers for monitoring congestion. However, their utility as decongestion biomarkers is limited by the fact that small changes may be caused by other conditions, and that they do not reflect the absolute change in plasma volume [42].

## 4. Biomarkers in HFrEF and in HFpEF

Unimportant differences in the prognostic value of biomarkers in HFpEF vs. HFrEF were found in previous studies except for cystatin-C and NT-proBNP; in fact, these two markers showed a less evident prognostic value in HFpEF [43].

Patients with HFpEF and HFrEF had higher median levels of hs-TnT, GDF-15 (growth differentiation factor 15), and NT-pro-BNP compared with controls, but not ST2; NT-proBNP and hs-TnT levels were higher in HFrEF in comparison with HfpEF [44]. In a former study [45], normal BNP values were present in 29% of symptomatic patients with HFpEF who had elevated pulmonary capillary wedge pressures; therefore, normal BNP values may be insufficient to rule out the diagnosis of HFpEF.

NPs are usually only moderately elevated in HFpEF patients, and levels may decrease in the asymptomatic phase. The reason for this is that NPs are liberated and produced in response to augmented myocardial wall stress. Hypertrophic hearts (typical in HFpEF) are not characterized by severely elevated end-diastolic wall stress. In contrast, in the case of supraventricular tachycardia or excess water (as in HFrEF), levels of NPs may become very high. In the first scenario, the lack of chronically increased wall stress causes lower NP production and lower circulating levels when compared to HFrEF. NP plasma levels increase in parallel with the severity of diastolic abnormalities [46].

## 5. Diagnostic, Prognostic and Therapeutic Significance of Common Congestion Biomarkers

Congestion in HF can be defined as fluid accumulation inside the intravascular compartment and the interstitial space because of increased cardiac filling pressures determined by maladaptive sodium and water retention by the kidneys [3]. It is the main reason for hospitalization in patients with ADHF, even if the severity of congestion varies widely between patients [6]. In a large, long-term European registry, 83% of all patients admitted to hospital with ADHF had clinical signs or symptoms of congestion [47].

The fluid accumulation leading to ADHF starts in the intravascular compartment. Continuously increased hydrostatic pressures in the capillary vessels subsequently lead to tissue congestion. Most patients with ADHF have a combination of both intravascular and tissue congestion [3].

Currently, the gold standard for assessing intravascular congestion is to measure right atrial pressure (normally 2–6 mmHg) and pulmonary artery systolic pressure (PASP; normally 3–8 mmHg) by right heart catheterization. However, right heart catheterization is invasive, and, therefore, routine performance of this procedure is not attractive. To assess changes in congestion on a day-to-day basis, non-invasive measurements are needed, such as changes in plasma levels of congestion biomarkers between admission to and discharge from the hospital.

Congestion biomarkers could be useful to identify subclinical congestion in hospitalized patients, with prognostic implications. In fact, incomplete decongestion at discharge from the hospital is associated with higher rates of both death and re-admission to the hospital for heart failure. Patients with residual congestion on day 7 of hospitalization have a more than two-fold increase in 180-day mortality and an almost two-fold increase in the risk of re-hospitalization for HF compared with patients without congestion [6]. It is therefore critical to identify patients with incomplete and subclinical congestion, and panels of congestion biomarkers may be useful for this purpose [48]. In this context, an analysis of ADHERE found that a multi-biomarker strategy for the assessment of patients hospitalized with HF added synergistic information about in-hospital mortality [49]. Moreover, biomarkers measured during inpatient treatment could be used to inform decisions about the timing of hospital discharge and the required intensity of post-discharge follow-up [50]. Observational data clearly demonstrate that the relationship between elevated NP levels at the time of AHF presentation and the level at discharge is closely associated with subsequent risk [51]. Fewer data are available on the relationship between changes in NP levels during HF hospitalization and subsequent events. Early observational studies in single-center cohorts identified either absolute discharge NP levels or the relative decrease in NPs during hospitalization as being predictive of post-discharge outcomes [52,53]. Although there are substantial variations among specific studies, generally, values of NPs at the time of hospital discharge have been found to be more predictive of post-discharge events than earlier values (or the change in values) that occur during hospitalization [50].

Multi-biomarker diagnostic approaches could be useful to predict outcomes in patients at risk or with overt HF in many settings. In the past, multi-biomarker scores have been evaluated for risk stratification in ADHF [54] and chronic HF [55], and for risk prediction of incident HF in the general population [56,57]. For example, Aimo et al. assessed the measurement of NT-proBNP and hs-TnT with prognostic thresholds stratified by GFR for risk prediction in chronic HF [58]. Many other multi-biomarker strategies have been proposed [50]. In most studies, the biomarkers were arbitrarily chosen. In contrast, in a sub-analysis of the Treatment of Preserved Cardiac Function Heart Failure with an Aldosterone Antagonist (TOPCAT) study, a machine-learning approach was applied to generate a multi-biomarker panel (from 49 available analytes) capable of predicting a composite of all-cause death or HF hospitalization in patients with HFpEF [59].

In recent years, diagnostic strategies based on multi-biomarkers reflecting different pathophysiological pathways have been proposed, with the aim of better understanding the phenotype of the disease in each individual patient and possibly designing an adapted therapy. For example, demonstrating an increase in a specific biomarker could lead to the initiation or titration of a therapy that neutralizes the mechanisms leading to such an elevation. Importantly, these strategies have never been tested in randomized controlled trials [48].

Further research is required to explore the use of a multi-biomarker approach for improving the diagnostic and prognostic precision of models, as well as to better understand disease phenotypes and direct the patient toward the best therapeutic strategy. The role of congestion biomarkers in the prognosis of ADHF would provide useful information about patients who may respond to more aggressive treatments and those who will progress and/or may become candidates for cardiac transplantation in the future. In addition, more research is needed to determine if new biomarkers can be used as a substitute for clinical trial results.

NPs may also guide therapy in HF, as serial NP measurements provide data about medication adjustments to achieve targets independently of symptoms. However, in this regard, the data seem quite conflicting. In patients with HFrEF, NP-guided therapy seems to be helpful. The studies STARS-BNP [60] and PROTECT [61] showed a decrease in cardiac events with NP-guided therapy. Furthermore, the mortality in patients aged <75 years decreased in the BATTLESCARRED [62] and TIME-CHF [63] studies, and in a meta-analysis. On the contrary, no differences were observed in the studies PRIMA [64] and GUIDE-IT [65]. In HFpEF patients and in the acute setting, no differences were demonstrated with NP-guided therapy compared with the conventional approach.

## 6. Conditions Other than Congestion with Increased Congestion Biomarkers (Figure 1)

NPs represent the gold standard for biomarkers in HF, and all major societies, including AHA and ESC, recommend the dosage of BNP and NT-proBNP for diagnosing HF in their guidelines. Nevertheless, there are determinants influencing the clinical interpretation of their levels. While obesity lowers BNP and NT-proBNP serum levels (adipocytes express the NP clearance receptors-C, NPR-C, causing researchers to believe this is the basis for low serum levels in obese people) [66], factors such as age, heart muscle diseases (hypertrophic cardiopathy, infiltrative myocardiopathies, Takotsubo syndrome, myocarditis, coronary artery disease), heart valve disorders, atrial fibrillation and flutter, cardiotoxic drugs, renal failure, anemia, critical illnesses (bacterial sepsis, burns, ARDS), stroke, and right-heart diseases (pulmonary embolism, pulmonary hypertension, congenital heart diseases) increase their plasmatic concentrations [67]. This happens because BNP is essentially secreted by ventricles as a response to left ventricular stretching or wall tension [68].

**Figure 1:**
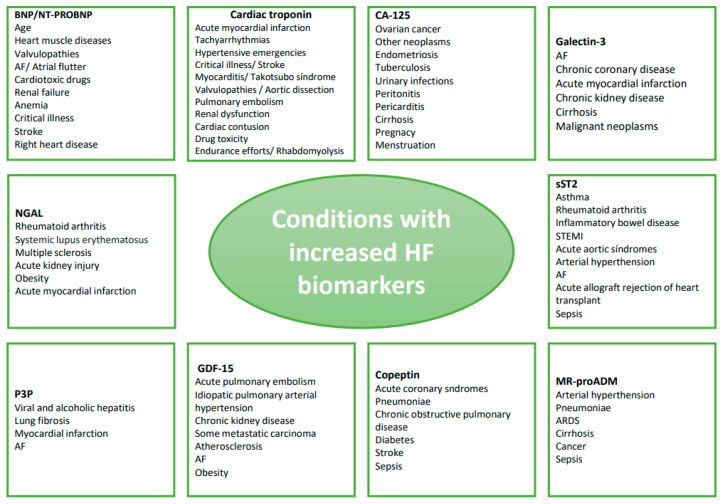
Conditions other than congestion with increased congestion biomarkers. BNP: Brain natriuretic peptide; NT-pro-BNP: N-terminal pro-B-type natriuretic peptide; AF: atrial fibrillation; STEMI: ST-elevation myocardial infarction; ARDS: acute respiratory distress syndrome; MR-proADM: Mid-regional Pro-Adrenomedullin; P3P: serum type III procollagen peptide; NGAL: neutrophil gelatinase-associated lipocalin; GDF-15: Growth Differentiation Factor 15; sST2: soluble suppression of tumorigenesis-2; CA-125: cancer antigen-125.

Cardiac troponins are traditionally used for the diagnosis of acute myocardial infarction (AMI), but they are also elevated in HF because of coronary causes, cytotoxicity, apoptosis, and inflammation. Troponin kinetics help distinguish between AMI and HF.

Other pathologies are characterized by cardiac troponin elevation: tachyarrhythmias, hypertensive emergencies, critical illnesses (shock/sepsis/burns), myocarditis, Takotsubo syndrome, valvular heart diseases, aortic dissection, pulmonary embolism, pulmonary hypertension, renal dysfunction, acute neurological events, cardiac contusion or cardiac procedures, thyroid disorders, infiltrative diseases, drug toxicity, extreme endurance efforts, or rhabdomyolysis [69].

In patients with COVID-19 pneumonia, there are high troponin and liver enzymes levels in many patients regardless of HF, which means that their diagnostic value in this context is low for separating these two entities [70]. On the other hand, these basic and very available biomarkers could be used in diagnosis of concurrent cardiac disease, such as HFrEF, as presented in the following paper [71].

In all these cases, an increase in cardiac troponin concentration in blood serum indicates reversible or irreversible damage to cardiomyocytes, and in some cases (impaired renal filtration, false-positive results, for example, due to the interaction of anti-cTnT and anti-cTnI antibodies with skeletal troponins released from muscle fibers in myopathies and rhabdomyolysis), elevation can occur even in the absence of cardiomyocyte damage [72].

Recent evidence about CA-125 suggests that plasma levels of this glycoprotein could be useful as biomarkers in HF, reflecting mesothelium activation due to hydrostatic pressure and inflammation. Apart from its common use as a tumor marker in ovarian cancer, an abnormally high expression of serum CA-125 is also present in other neoplasms (breast, lung, uterus, and stomach, lymphangioleiomyomatosis) [73], in non-malignant pathologies (endometriosis, tuberculosis, urinary infections, peritonitis, pericarditis, cirrhosis), and physiological conditions (pregnancy, menstruation) [74].

Galectin-3 is widely expressed in human tissues, including all types of immune cells, epithelial cells, endothelial cells, and sensory neurons. It contributes to cell survival, cell–cell interaction, and cell-extracellular matrix interaction; thus, it is involved in cell differentiation, inflammation, fibrogenesis, angiogenesis, and host defense [75].

Its serum levels are elevated in many other conditions: atrial fibrillation (particularly in persistent AF), chronic coronary heart disease, AMI, chronic kidney disease, cirrhosis, renal cell carcinoma and other malignant lesions such as hepatocellular carcinoma, prostate cancer, pancreatic cancer, colorectal cancer, bladder cancer and lymphoma.

sST2 has gained interest as a potential biomarker for inflammatory diseases like asthma, rheumatoid arthritis, and inflammatory bowel disease, as well as in cardiovascular pathophysiology. It can be used for predicting and monitoring HF; in fact, it was included in the 2017 ACC/AHA update of HF guidelines. Elevated levels of serum sST2 have been reported in AMI, acute aortic syndromes, arterial hypertension, atrial fibrillation, and in heart transplant recipients with acute allograft rejection [76]. Lastly, a prognostic role for this biomarker has been reported in septic patients [77]. The source of sST2 is not well established, being hypothesized to be both a cardiac fibroblast/cardiomyocyte production in response to injury or stress and a non-myocardial production from endothelial cells; however, the effect of its release is a reduction of the cardio-protective effect of the cytokine IL-33 in situations of biomechanical cardiac stress [78].

Mid-regional pro-adrenomedullin (MR-proADM) represents the level and activity of adrenomedullin. Its synthesis is widely distributed in tissues, including bone, adrenal cortex, kidney, lung, blood vessels, heart, adipose tissue, anterior pituitary gland, thalamus, and hypothalamus; among its effects are vasodilatation, positive inotropy, diuresis, bronchodilation, and anti-inflammatory/anti-microbial action. High levels of this biomarker have been reported in HF, hypertension, pneumoniae, ARDS, cirrhosis, and cancer. Moreover, it has shown effectiveness as a diagnostic marker for differentiating between septic and non-septic patients with Systemic Inflammatory Response Syndrome (SIRS) and as a prognostic marker for stratifying the mortality risk in patients with sepsis [79]; recently, it has also been demonstrated to predict mortality in critically ill COVID-19 patients [80].

Copeptin, a surrogate marker of vasopressin (AVP), has an application in the diagnosis of diabetes insipidus and other diseases with altered vasopressin secretion. High copeptin serum levels could have a diagnostic and prognostic role in acute coronary syndromes, a prognostic value in pneumoniae, chronic obstructive pulmonary disease, diabetes mellitus, stroke, and sepsis [81], and AVP may be involved in the development of the metabolic syndrome. In fact, AVP has several actions, which include vasoconstriction, platelet aggregation, stimulation of glycogenolysis in the liver, effects on lipid metabolism, and the release of insulin and glucagon from the pancreas [82].

GDF-15 is a stress-responsive cytokine, widely present in mammalian tissues and plays multiple roles in various pathologies, performing anti-inflammatory and immunosuppressive actions. Elevated circulating levels of GDF-15 have been reported in patients with acute pulmonary embolism, idiopathic pulmonary arterial hypertension, chronic kidney disease, metastatic prostate, breast, and colorectal carcinoma, with a strong association between serum levels, histological malignant grade, and metastatic progression. As regards CV diseases, GDF-15 is related to cardiometabolic risk, and its synthesis increases during tissue injuries and inflammatory states. High levels of GDF-15 are associated with the development and progression of pathologies such as HF, atherosclerosis, atrial fibrillation, obesity, and diabetes [83].

P3P serum type III procollagen peptide is released by fibroblasts during collagen synthesis and indicates a rapid collagen turnover. Therefore, its serum levels are elevated in viral and alcoholic hepatitis, lung fibrosis [84], CHF [85], post AMI, and atrial fibrillation [86]. It has been hypothesized that P3P elevation could reflect myocardial fibrotic scar formation after an ischemic cardiac event and atrial fibrosis in atrial fibrillation patients. In acute HF, high levels of P3P are associated with adverse outcomes and are linked to acute heart remodeling and congestive hepatic damage.

The neutrophil gelatinase-associated lipocalin (NGAL), among the members of the adipokines superfamily, has emerged as a pleiotropic acute phase protein that takes part in many inflammatory, immune, and metabolic processes; thus, it has a potential role as a biomarker in these fields. Its serum level is upregulated in several acute and chronic inflammatory/autoimmune conditions (rheumatoid arthritis, systemic lupus erythematosus, multiple sclerosis, acute kidney injury) [87], as well as in some cardiovascular pathologies: obesity, AMI [88], and AHF, where NGAL appears to be a marker of renal dysfunction and a useful tool to identify patients at the early stages of Cardio-Renal Syndrome (CRS) [89].

In clinical medicine, the BUN/Cr ratio has been extensively used to differentiate pre-renal kidney disfunction from intrinsic renal parenchymal disease. The BUN/Cr ratio is often used to determine dehydration in patients presenting to emergency departments, and a high ratio has a prognostic value in many diseases, including stroke [90], gastrointestinal bleeding, hip fracture, and HF (an elevated admission BUN/Cr identifies patients with HF-induced renal dysfunction) [91].

## 7. Novel Congestion Biomarkers and Future Prospects

Congestion is the principal cause of HF decompensation and hospitalization, leading to a worst prognosis. Therefore, the early detection of subclinical congestion with new biomarkers is very important for the management of these patients.

Most new biomarkers are not used daily for the diagnosis of HF or for differential diagnosis in patients with new dyspnea. New biomarkers are usually used for prognostic stratification in HF. They may add prognostic information to common cardiac biomarkers.

Cardiac interstitial fibrosis markers seem to have the biggest prognostic value in HF patients [92]. Among the new biomarkers, Galectin-3, GDF-15, and Copeptin are always the most reliable in prognostic stratification in HF.

As an HF biomarker, Galectin-3 may have a diagnostic and prognostic role (prognostic in both acute and chronic HF), since its expression is upregulated, especially in severe HF patients and in those with renal dysfunction. To date, Galectin-3 measurement is recommended by the 2017 Guidelines of the American Heart Association for risk stratification and prognosis evaluation of patients with HF [33].

GDF-15 levels are slowly increasing over time in patients with CHF, and these increases are associated with worsening functional status and adverse outcomes [93]. Moreover, high GDF-15 levels are associated with an increased risk of developing adverse left ventricular reshaping and HF following an episode of acute coronary syndrome (ACS) [94]. GDF-15 adds predictive information to clinical risk factors and cardiac biomarkers, including BNP (NT-proBNP) [95]. The index of [(CRP + GDF-15s + sST2)/NT-proBNP] may be useful to discriminate HFpEF from HFrEF [96].

Fibroblast growth factor-21 (FGF21) appears to be a promising biomarker candidate for HF. Pre-clinical studies have shown that FGF21 is implicated in the physiopathology of HF (oxidative stress, cardiac hypertrophy, and inflammation of cardiomyocytes). However, in the clinical setting, FGF21 levels seem to rise in HF. It is unclear whether FGF21 might have a causal role in HF, and whether circulating FGF21 can be used as a biomarker to improve the prediction, diagnosis, and prognosis of HF [97].

Fibroblast growth factor-23 is a hormone dominantly secreted by osteocytes and osteoblasts in bones; however, it can also be produced by liver and heart muscle under stress [98]. It showed an association between elevated FGF-23 and left ventricular hypertrophy [94]. In patients with new-onset and worsening HF, higher FGF23 concentrations were independently associated with volume overload, less successful uptitration of ACEi/ARBs, and an increased risk of all-cause mortality and HF hospitalization [99].

Copeptin has been found to be an excellent marker of HF development in patients with ACS, in the risk stratification of patients with HF symptoms presenting acutely to an emergency unit, as well as an accurate marker of risk in both hospitalized and ambulatory patients with CHF [100]. As regards HF, elevated levels of copeptin are associated with all-cause mortality in HF patients, with results comparable with NT-proBNP prognostic value [101].

Neutrophil gelatinase-associated lipocalin (NGAL) is a promising diagnostic biomarker. It is a human protein belonging to the lipocalin superfamily. Both plasma and urinary NGAL levels are increased in HF patients. The elevation is dependent on the clinical stage of HF, together with the presence of renal failure and consequent congestion [91].

Widely recognized as a major marker of neuroendocrine tumor (NET), chromogranin A (CgA) has been found to be related to the clinical deterioration and higher risk of mortality in patients with AHF and CHF [102]. High levels of CgA were independently related to 1-year death and hospitalization for HF [103].

Another molecule, fatty acid-binding protein 4 (FABP4), has recently been shown to play a role in atherosclerosis and coronary artery disease, and it has been directly related to cardiac alterations such as left ventricular hypertrophy (LVH) and both systolic and diastolic cardiac dysfunction [104]. Some recent studies showed the increase of FABP4 levels in HF patients and propose it as a biomarker for congestion [105].

The P3P has been defined as a marker for fibrosis in patients with hepatic disease [106,107]. A high P3P level during AHF served as a comprehensive biomarker of liver dysfunction with volume overload and renal dysfunction. A high P3P level at admission may be able to predict adverse outcomes and can be a marker for congestion in HF patients [84].

Elevated soluble neprilysin (sNEP) levels have been suggested as a prognostic biomarker in HFrEF. Levels of sNEP were significantly reduced in HFrEF when compared to the controls [108].

Recently, four key genes (NSG1, NPPB, PHLDA1, and SERPINE2) were identified as potential biomarkers for HF, providing a new research idea for the treatment of HF [109].

N-acetylglucosamine/galactosamine (GlycA) and sialic acid (GlycB) correlate with inflammation status and with prognostic value in long-term outcomes of patients with HF [110].

Researchers are searching for new biomarkers with possible implications for diagnosis, risk stratification, and treatment effect monitoring. These biomarkers could potentially be used to better define study inclusion criteria and enable the enrolment of patients who are more likely to respond to a specific drug. In the case of a close relationship between biomarker levels and clinical endpoints, changes in biomarker levels after a treatment may be a surrogate endpoint, potentially reducing the duration and cost of a clinical trial. With omics-based tools, biomarkers might be more precisely selected for use in clinical trials to identify responses that closely reflect the biological effects of the new drug. The future role of biomarkers will be linked to providing a distinct phenotype for HF patients in order to choose the right therapy. Old and new HF biomarkers could be integrated into validated scores for prognostic stratification [111] and linked to new pharmacological targets [112].

However, the new biomarkers still need to be validated for clinical implications in multicentric studies with large populations. Further studies are needed prior to formalizing individual patient care algorithms guided by fibrosis biomarkers. Therapy guidance in chronic HF using galectin-3 and sST2, was suggested by several post hoc analyses [113].

There are emerging data about sST2, galectin-3, and procollagen as potential uses for guiding therapies. Mortality benefits from statin use, as well as anti-fibrotic therapies, such as aldosterone antagonism, may vary based on levels of these fibrosis markers [114].

**Figure 2:**
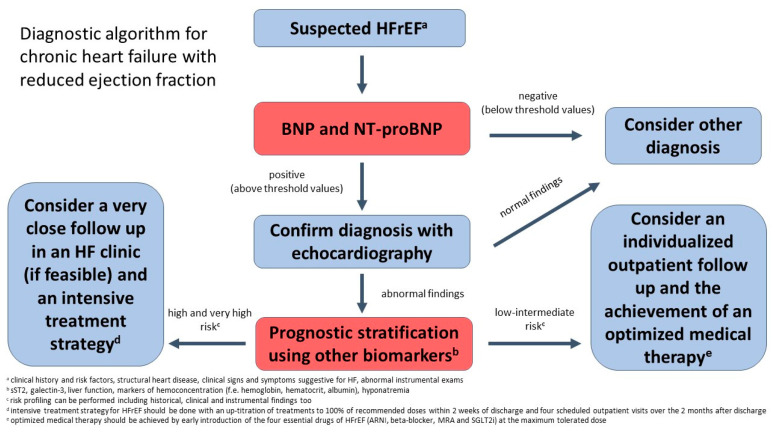
Diagnostic algorhitm for chronic Heart Failure with reduced ejection fraction. HF: Heart Failure; HFrEF: Heart Failure with reduced ejection fraction; BNP: Brain natriuretic peptide; NT-pro-BNP: N-terminal pro-B-type natriuretic peptide.

We propose an algorithm for the diagnosis and risk stratification of patients with suspected HF (Figure 2). In patients presenting dyspnea, measurement of BNP or NT-proBNP may be useful for excluding HF in ambulatory and emergency department settings. If an increase in these peptides is detected, it allows for the confirmation of the HFrEF diagnosis by echocardiographic evaluation. Once an HF diagnosis is reached, biomarkers may be useful for prognostic stratification too. In fact, a widening array of biomarkers, including markers of myocardial injury, inflammation, oxidative stress, vascular dysfunction, and matrix remodeling have been shown to provide incremental prognostic information over natriuretic peptides. These may allow to for the identification of high- and very-high risk patients, in which it would be preferable to start a very close outpatient follow-up, by referring the patient to an HF clinic and to pursue an intensive treatment strategy, such as the one tested in the STRONG-HF trial [115].

In the future, it will be very interesting to see how new drugs for HF affect congestion biomarkers. Among the new drugs, gliflozins promote diuresis and natriuresis [116]. In 60 outpatients with CHF and T2D, empagliflozin initiation was associated with a significant decrease in CA125 levels without modifying the trajectory of NT-proBNP [117]. The authors hypothesized that empagliflozin might predominantly promote extravascular decongestion. However, in 59 consecutive patients with type 2 diabetes admitted for ADHF and randomized to empagliflozin vs. placebo seven days after randomization, the NT-proBNP level was significantly lower in the empagliflozin group than in the conventional glucose-lowering therapy group [118]. In patients with worsening HFrEF, vericiguat significantly decreased NT-proBNP levels compared with placebo [119]. 

To date, BNP is a substrate of neprilysin; BNP levels increase with neprilysin inhibition. So, the clinical validity of measuring BNP in sacubitril/valsartan-treated patients has been questioned, and the use of NT-proBNP has been preferred and recommended because it is not a substrate of neprilysin inhibition. NT-proBNP should be measured within 8 to 10 weeks of drug initiation [120].

Finally, new trials should be designed to combine different classes of diuretics/drugs to treat therapy-resistant edema in patients with HFrEF, and new biomarkers could play a role in the phenotyping of patients most sensitive to specific diuretics/drugs.

## 8. Conclusions

The search for cardiac biomarkers to enhance the prediction, diagnosis, and prognosis of HF is a major challenge in cardiology. Several biomarkers correlated with different findings of HF pathophysiology were studied. However, they still need to be validated for clinical implications. The most interesting role of biomarkers may be in helping the choice of the right therapy in HF patients by HF score and providing specific phenotypes of HF patients.

## Figures and Tables

**Table 1:** Established and emerging biomarkers in HF.

Main Group	Subgroup	Biomarker
Myocardial insult	Myocyte stretch	ANP, BNP, NT-proBNP, MR-proANP, GDF-15, neuregulin
	Myocardial injury	Troponin T, TRoponin I, hsTN, heart type fatty acid protein, myosin light-chain kinase1, creatinine kinase MB fraction
	Oxidative stress	Myeloperoxidase, MR-proADM, oxidized low-density lipoprotein, urinary biopyrrins, plasma malondialdehyde
Neurohormonal activation	Renin-Angiotensin System	Renin, Angiotensin II, Aldosterone
	Sympathetic Nervous System	Norepinephrine, Chromogranin A
	Arginine Vasopressin system	Arginine vasopressin, copeptine
	Endothelin	Endothelin-1, big proET-1
		Chromogranin A and B
Myocardial Remodeling	Inflammation	C-reactive protein, TNF-α, Fas (APO-1), interleukins 1, 6, and 18, cytokines, procalcitonin, adipokines, adiponectin
	Hypertrophy/Fibrosis	SolubleST2, Galectin-3, matrix metalloproteinases, collagenpeptide

ANP—atrial natriuretic peptide, BNP—B-type natriuretic peptide, GDF-15—growth differentiation factor 15, NT-proBNP—N-terminal pro B-type natriuretic peptide, MR-proANP—mid-regional pro atrial natriuretic peptide, MR-proADM—mid-regional pro-adrenomedullin, TNF-α—tumor necrosis factor 1.

## Data Availability

No new data were created.
